# Supplementary material for: Refolding and characterization of two G protein-coupled receptors purified from E. coli inclusion bodies
Source: PLoS One. 2021 Feb 24;16(2):e0247689. doi: 10.1371/journal.pone.0247689 (PMC7904181; doi:10.1371/journal.pone.0247689)

Raw data - Fig 1, “GPR3“

SDS-PAGE:

MW X F1 F2 F3 X X X X X

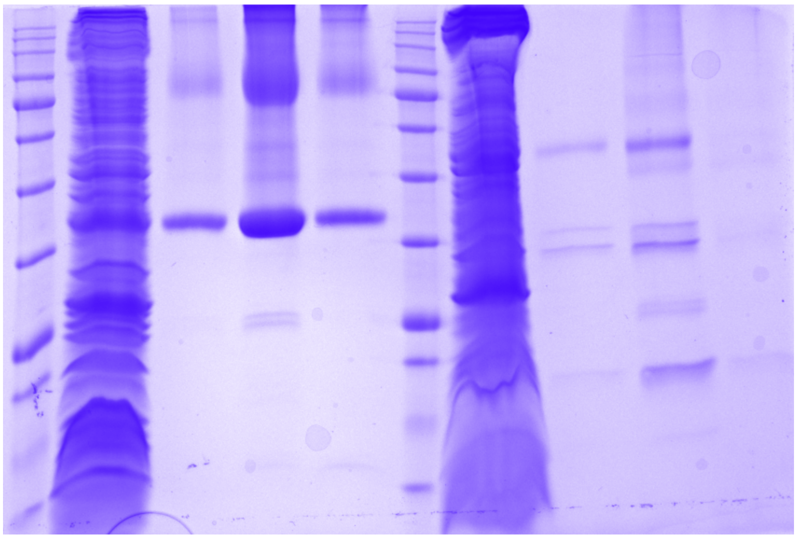

Western blot:

MW X X X F1 F2 F3 X

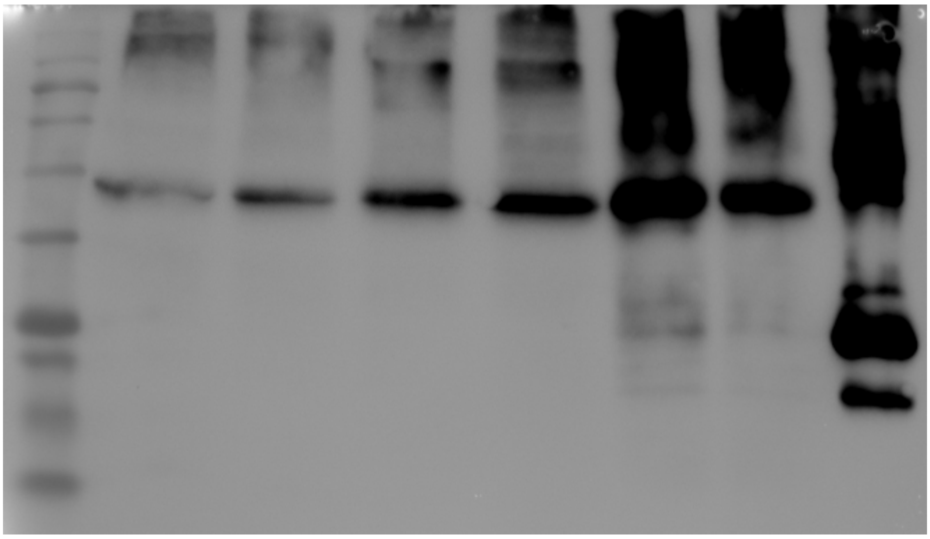

Raw data - Fig 1, “S1P1“

SDS-PAGE:

MW X X F1 F2 MW

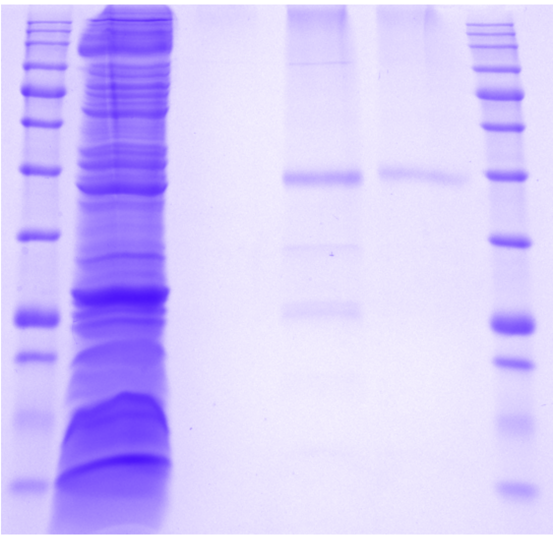

Western blot:

MW X X X X X X F1 F2 X

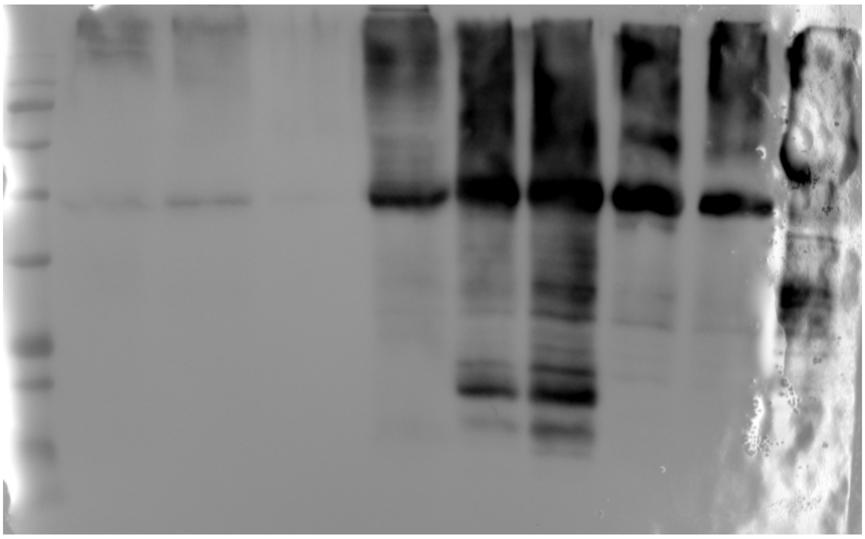

Raw data - Fig 5, “S1P1“

“Without lipid“

MW X X F1 F2 F3 X X X X

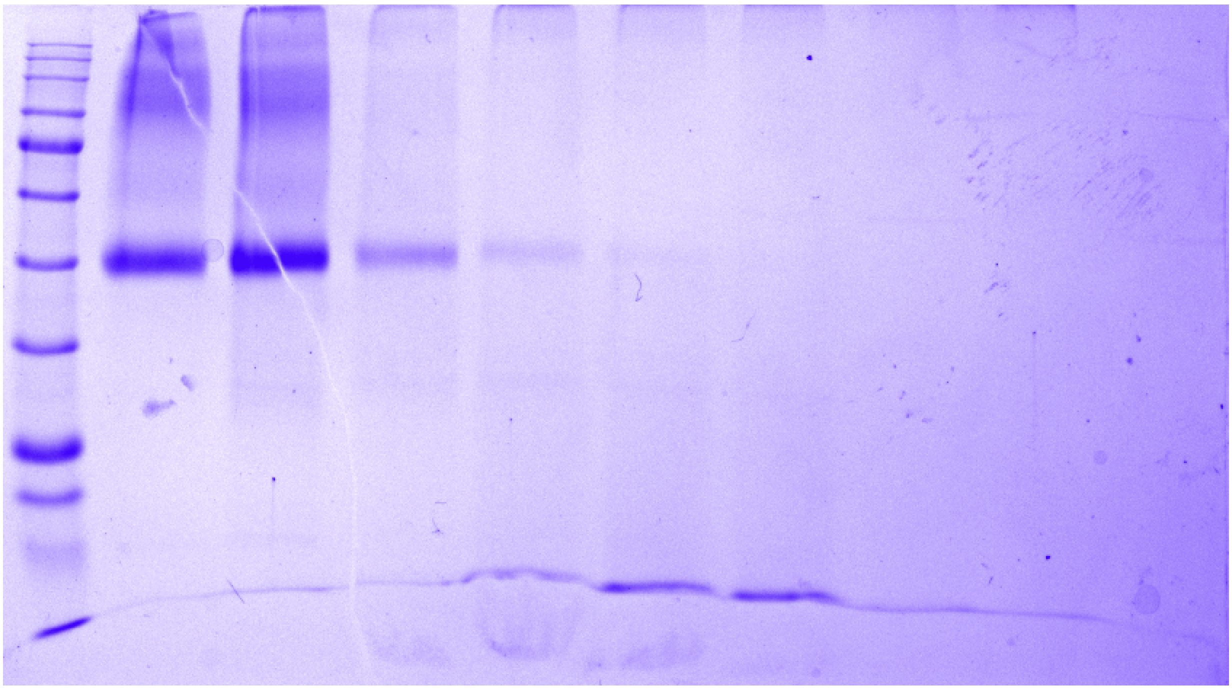

“With lipid“

MW F1 F2 F3 X X X X X X

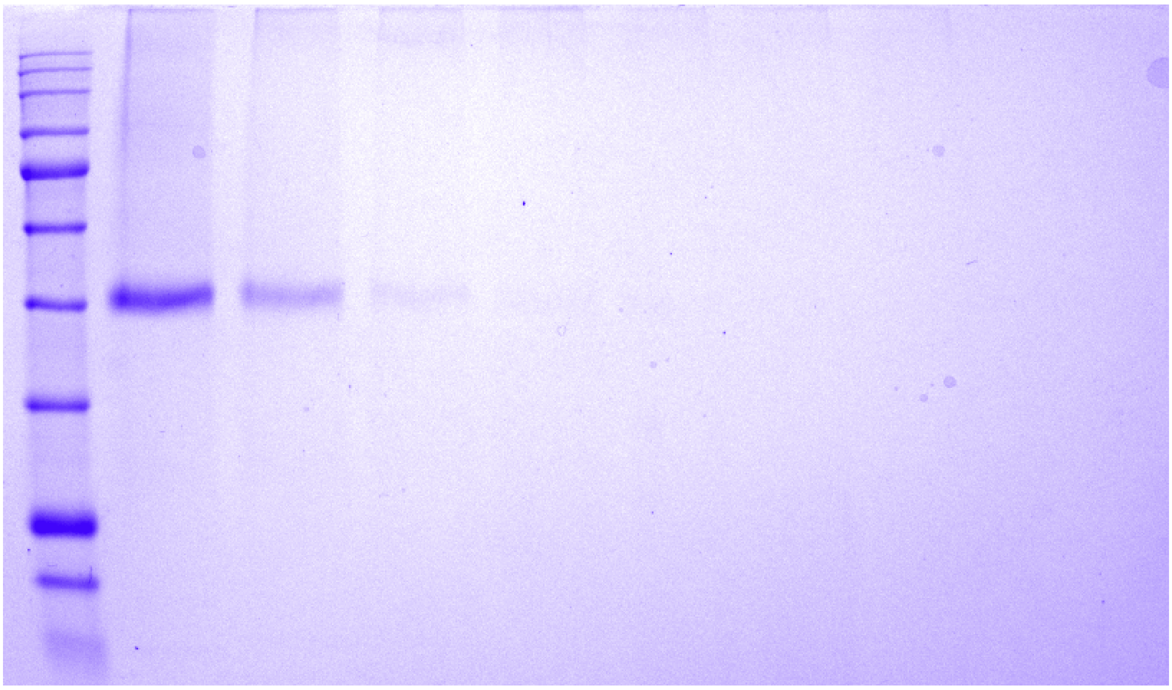

Raw data - Fig 5, “GPR3“

“Without lipid“

MW F1 F2 F3 F4 X X X X X

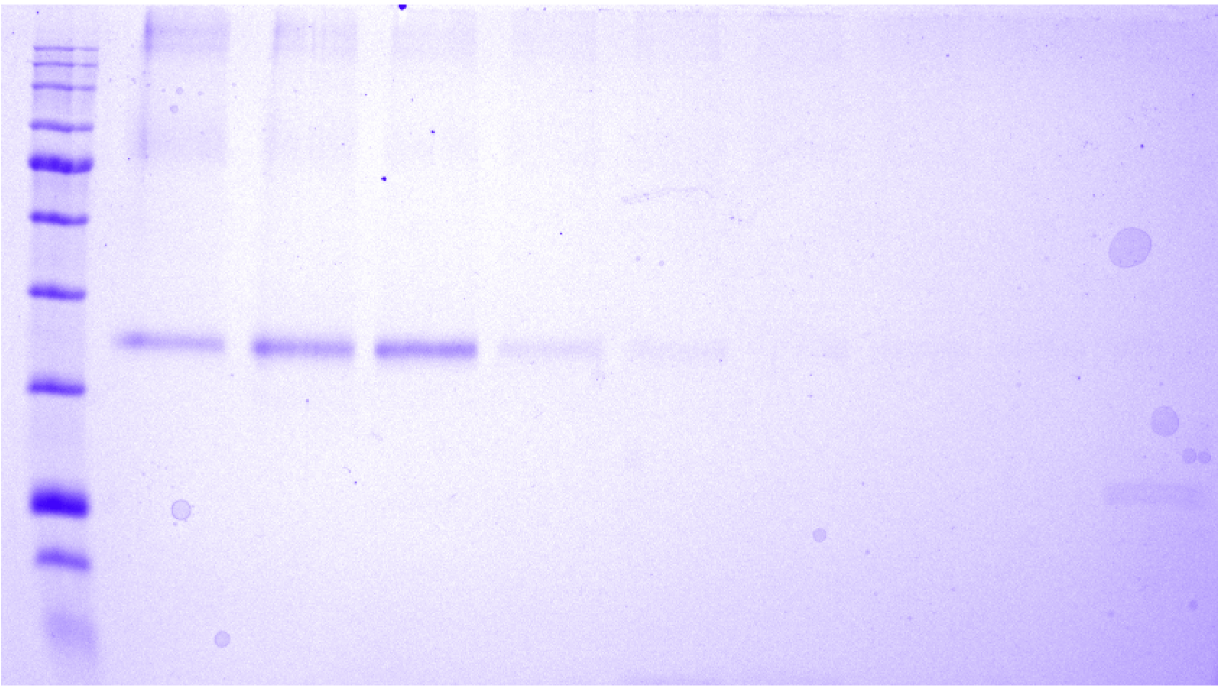

“With lipid“

MW F1 F2 F3 F4 F5 F6 F7 X X

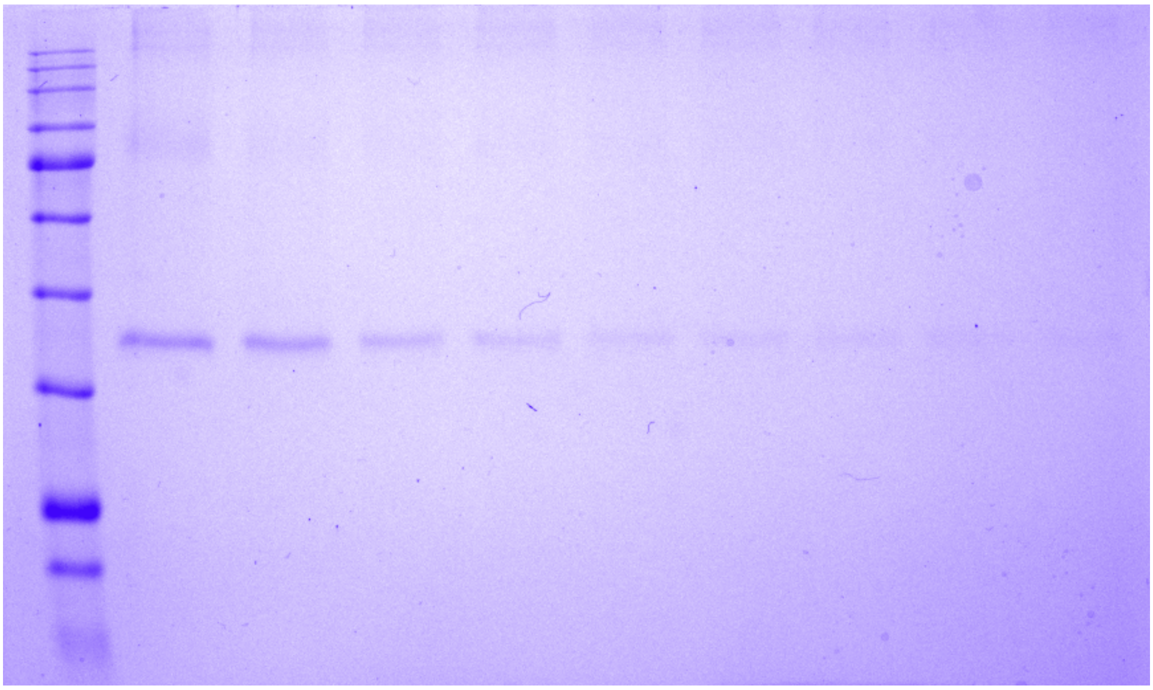

Supplement: S1 Raw images — (PDF) [file pone.0247689.s001.pdf]
