# Supplementary material for: Refolding and characterization of two G protein-coupled receptors purified from E. coli inclusion bodies
Source: PLoS One. 2021 Feb 24;16(2):e0247689. doi: 10.1371/journal.pone.0247689 (PMC7904181; doi:10.1371/journal.pone.0247689)
Supplement: S1 Table — Screens marked with a dot were used in crystallization attempts for S1P1 and GPR3. The crystallization screen according to [30] was prepared based on the published crystallization conditions. (DOCX) [file pone.0247689.s002.docx]

**S1 Table. Summary of all screening solutions used as well as an overview of the varying ingredients used in the crystallization attempts.** Screens marked with a dot were used in crystallization attempts for S1P_1_ and GPR3. The crystallization screen according to [30] was prepared based on the published crystallization conditions.

| **Screen** | **Manufacturer** | **S1P_1_** | **GPR3** | **Variation** |
| --- | --- | --- | --- | --- |
| Morpheus Screen | Molecular Dimension | ● | ● | complex screen |
| MemMeso Screen | Molecular Dimension | ● | ● | complex screen |
| Anion Screen | Qiagen | ● | ● | salt: anions |
| Cation Screen | Qiagen | ● | ● | salt: cations |
| Classic-I-Screen | Qiagen | ● | ● | salt; precipitants |
| Classic-II-Screen | Qiagen | ● | ● | salt; precipitants |
| PEG-Screen | Qiagen | ● | ● | salt; PEGs |
| PEG-II-Screen | Qiagen | ● | ● | salt; PEGs |
| pH-clear Screen | Qiagen | ● | ● | salt; pH |
| Screen according to [30] | Literature | ● | ● | salt; precipitants |
| pH-clear II Screen | Qiagen | - | ● | salt; pH; precipitants |
| Cryo Screen | Qiagen | - | ● | cryo solutions |
